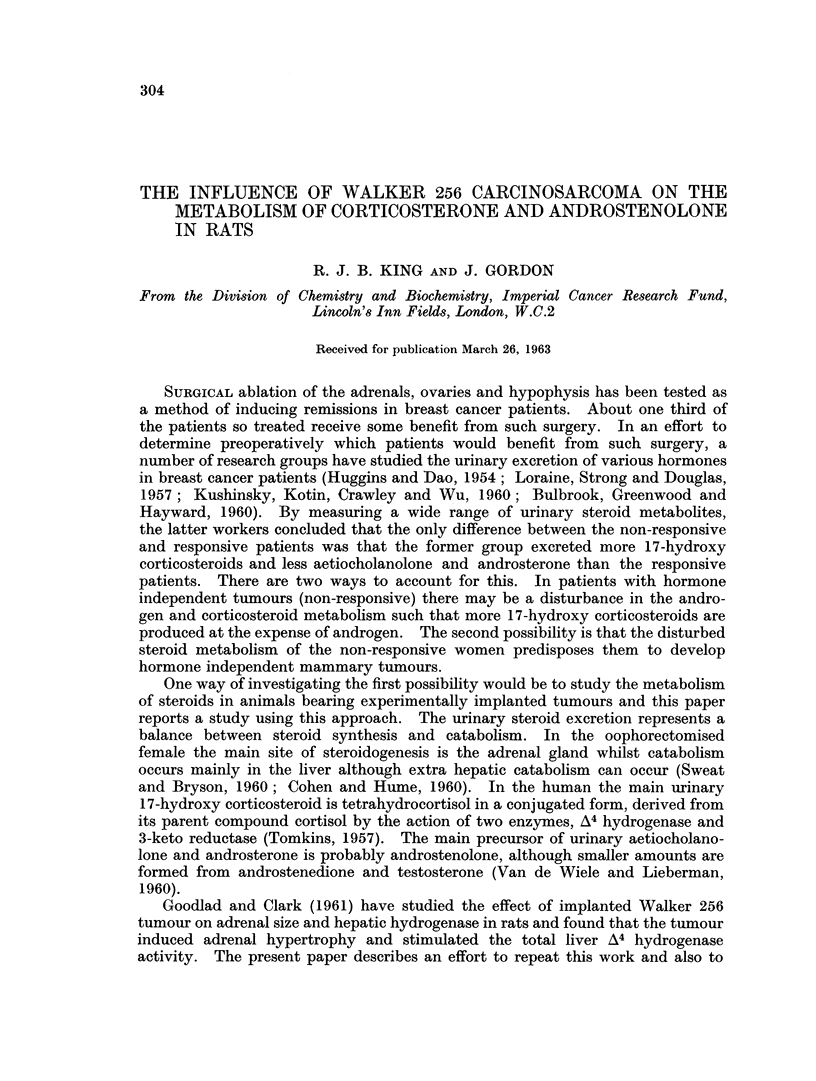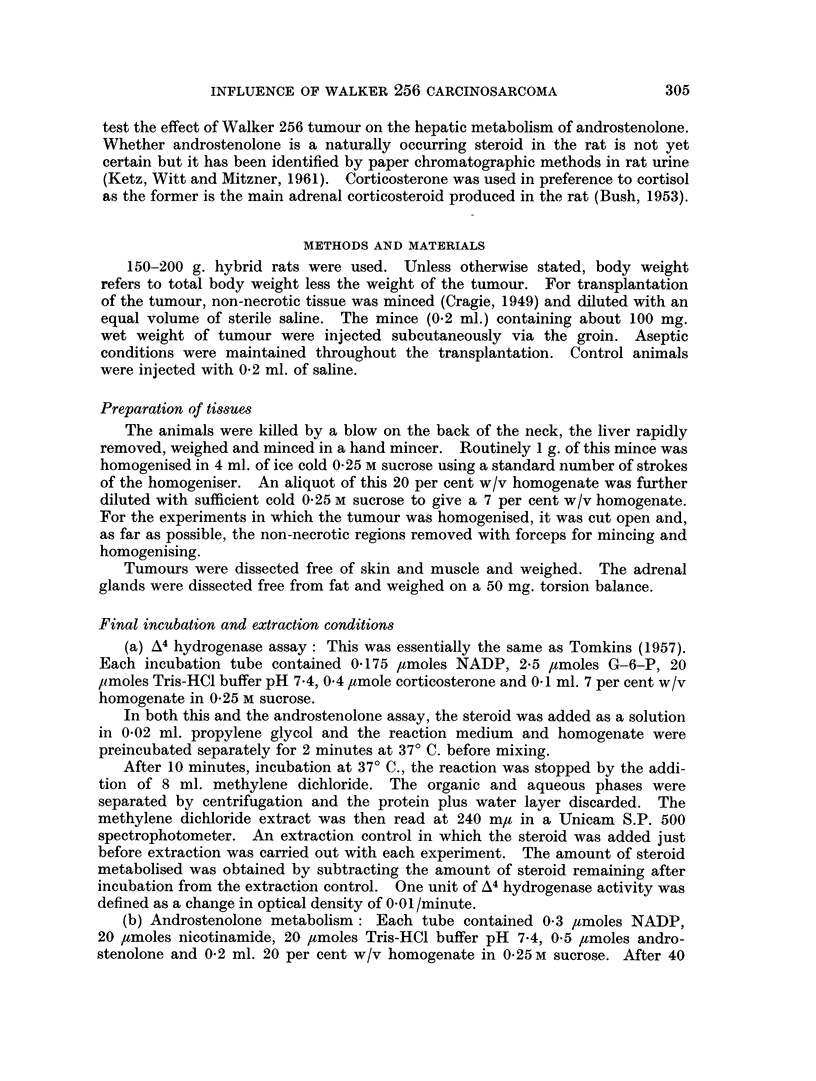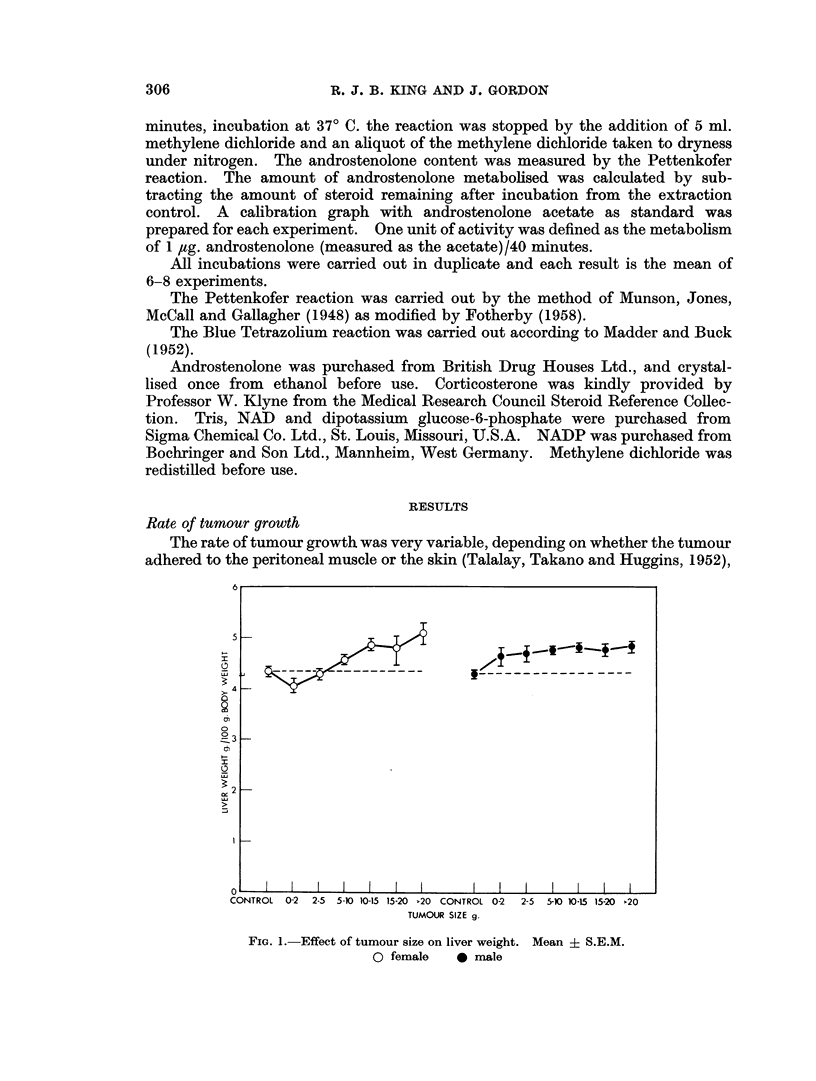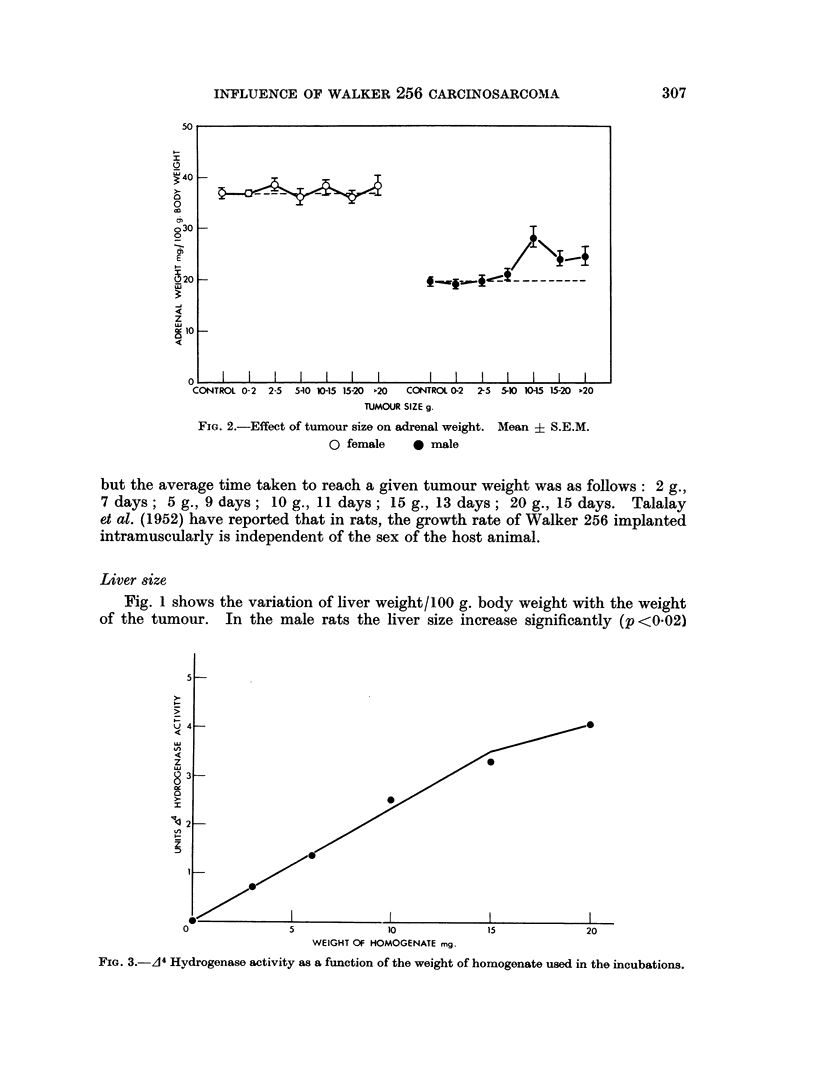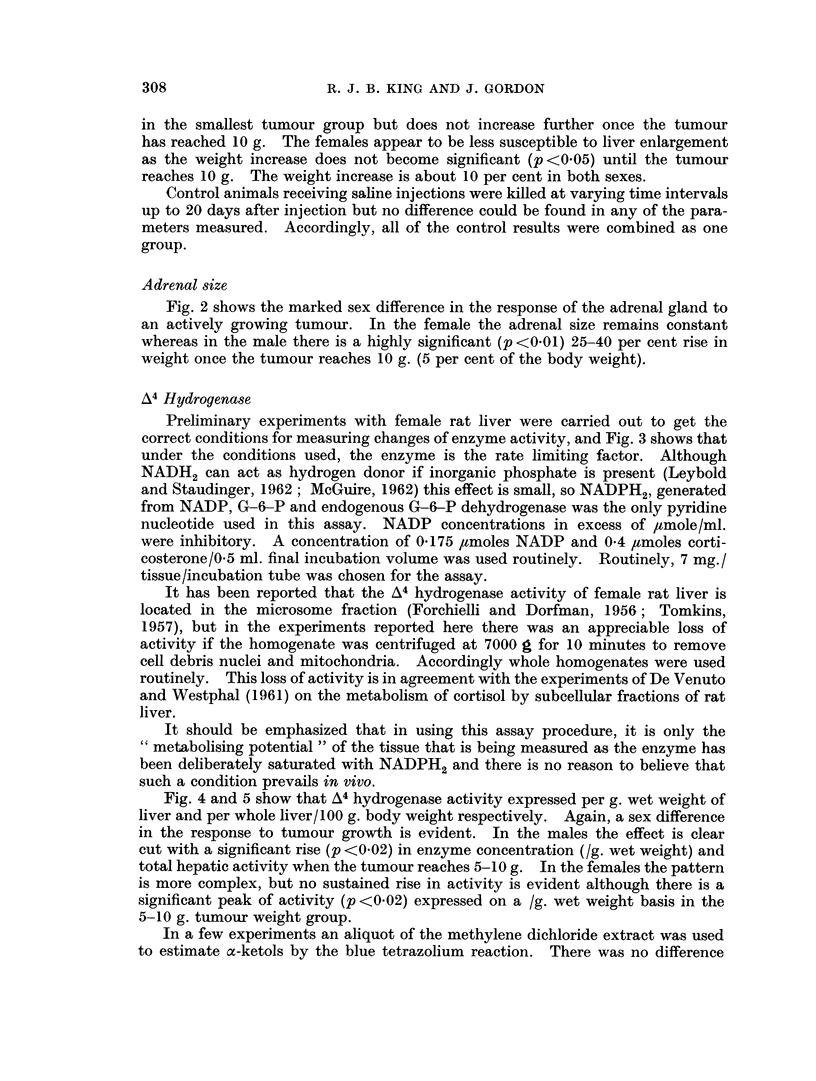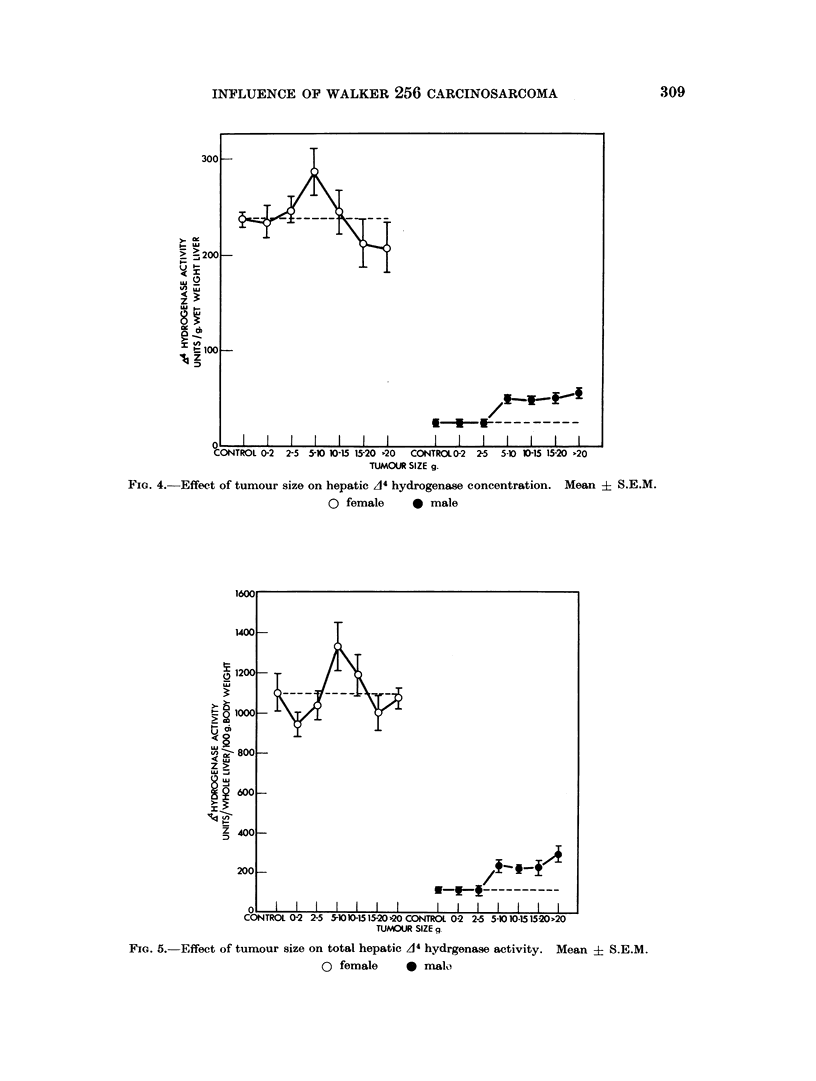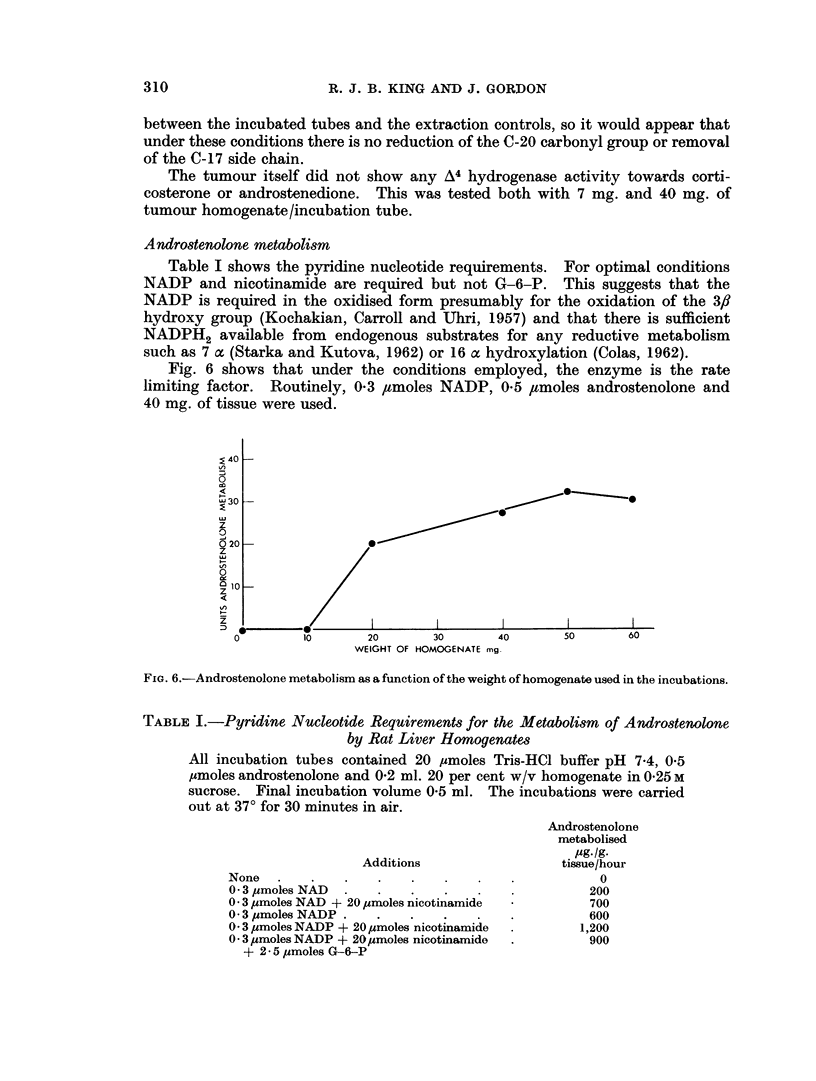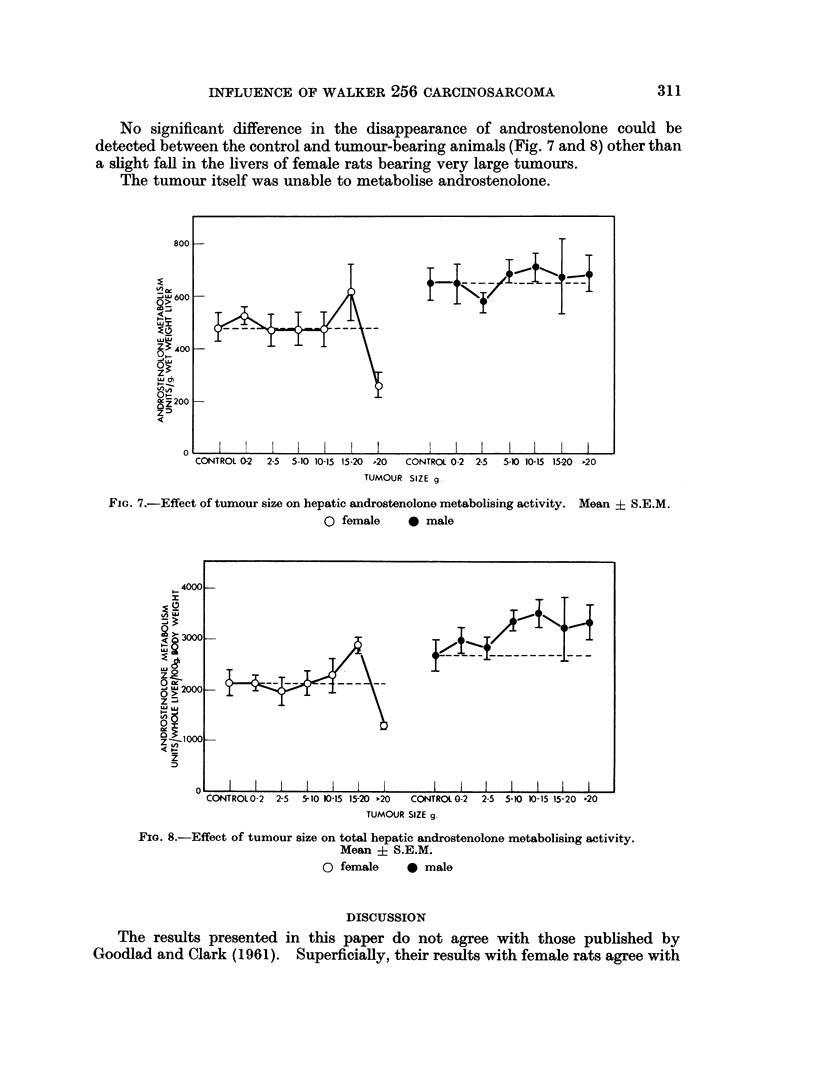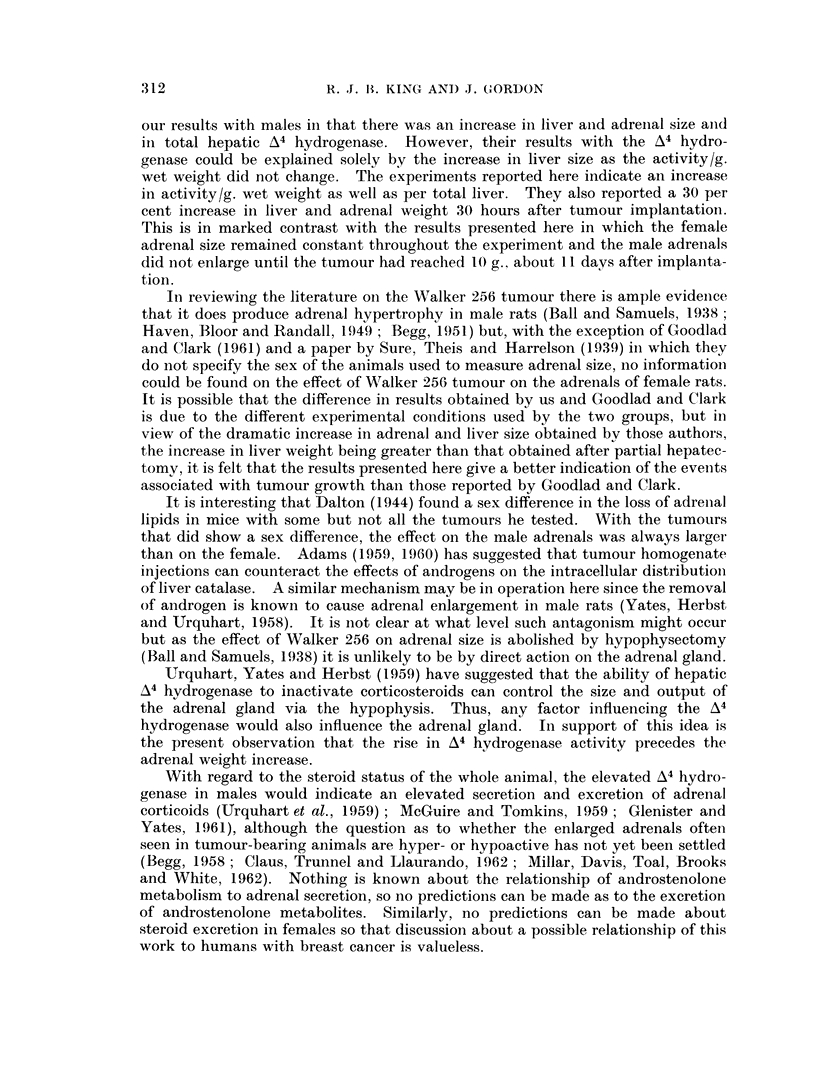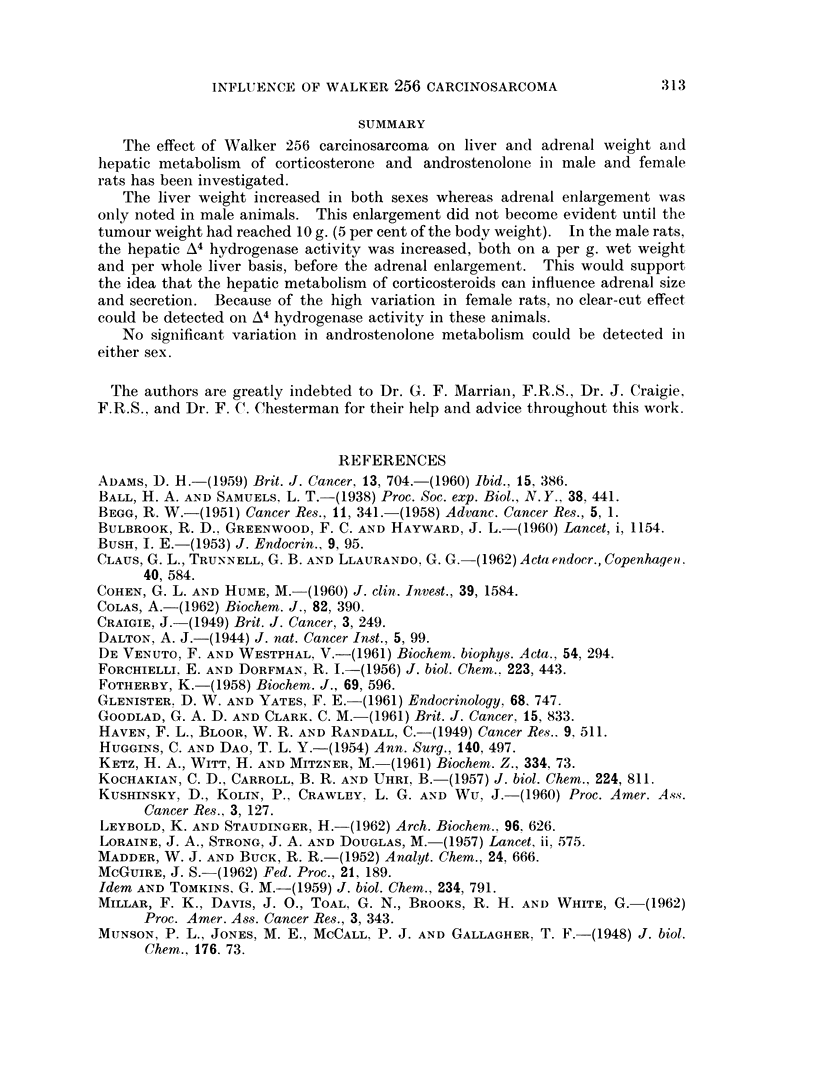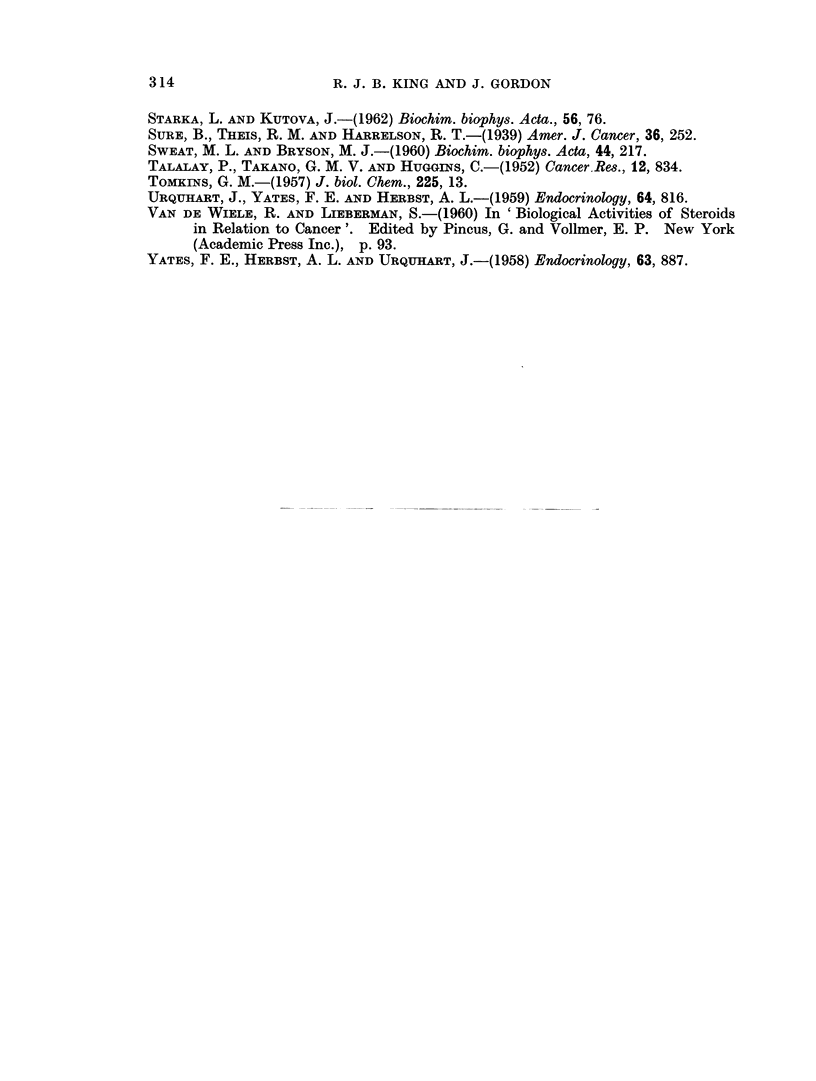# The Influence of Walker 256 Carcinosarcoma on the Metabolism of Corticosterone and Androstenolone in Rats

**DOI:** 10.1038/bjc.1963.44

**Published:** 1963-06

**Authors:** R. J. B. King, J. Gordon


### THE INFLUENCE OF WALKER 256 CARCINOSARCOMA ON THE METABOLISM OF CORTICOSTERONE AND ANDROSTENOLONE IN RATS

R. J. B. KING AND J. GORDON

*From the Division of Chemistry and Biochemistry, Imperial Cancer Research Fund, Lincoln's Inn Fields, London, W.C.2*

Received for publication March 26, 1963

SURGICAL ablation of the adrenals, ovaries and hypophysis has been tested as a method of inducing remissions in breast cancer patients. About one third of the patients so treated receive some benefit from such surgery. In an effort to determine preoperatively which patients would benefit from such surgery, a number of research groups have studied the urinary excretion of various hormones in breast cancer patients (Huggins and Dao, 1954 ; Loraine, Strong and Douglas, 1957 ; Kushinsky, Kotin, Crawley and Wu, 1960 ; Bulbrook, Greenwood and Hayward, 1960). By measuring a wide range of urinary steroid metabolites, the latter workers concluded that the only difference between the non-responsive and responsive patients was that the former group excreted more 17-hydroxy corticosteroids and less aetiocholanolone and androsterone than the responsive patients. There are two ways to account for this. In patients with hormone independent tumours (non-responsive) there may be a disturbance in the androgen and corticosteroid metabolism such that more 17-hydroxy corticosteroids are produced at the expense of androgen. The second possibility is that the disturbed steroid metabolism of the non-responsive women predisposes them to develop hormone independent mammary tumours.

One way of investigating the first possibility would be to study the metabolism of steroids in animals bearing experimentally implanted tumours and this paper reports a study using this approach. The urinary steroid excretion represents a balance between steroid synthesis and catabolism. In the oophorectomised female the main site of steroidogenesis is the adrenal gland whilst catabolism occurs mainly in the liver although extra hepatic catabolism can occur (Sweat and Bryson, 1960 ; Cohen and Hume, 1960). In the human the main urinary 17-hydroxy corticosteroid is tetrahydrocortisol in a conjugated form, derived from its parent compound cortisol by the action of two enzymes, $\Delta^4$ hydrogenase and 3-keto reductase (Tomkins, 1957). The main precursor of urinary aetiocholanolone and androsterone is probably androstenolone, although smaller amounts are formed from androstenedione and testosterone (Van de Wiele and Lieberman, 1960).

Goodlad and Clark (1961) have studied the effect of implanted Walker 256 tumour on adrenal size and hepatic hydrogenase in rats and found that the tumour induced adrenal hypertrophy and stimulated the total liver $\Delta^4$ hydrogenase activity. The present paper describes an effort to repeat this work and also to test the effect of Walker 256 tumour on the hepatic metabolism of androstenolone. Whether androstenolone is a naturally occurring steroid in the rat is not yet certain but it has been identified by paper chromatographic methods in rat urine (Ketz, Witt and Mitzner, 1961). Corticosterone was used in preference to cortisol as the former is the main adrenal corticosteroid produced in the rat (Bush, 1953).

### METHODS AND MATERIALS

150–200 g. hybrid rats were used. Unless otherwise stated, body weight refers to total body weight less the weight of the tumour. For transplantation of the tumour, non-necrotic tissue was minced (Cragie, 1949) and diluted with an equal volume of sterile saline. The mince (0·2 ml.) containing about 100 mg. wet weight of tumour were injected subcutaneously via the groin. Aseptic conditions were maintained throughout the transplantation. Control animals were injected with 0·2 ml. of saline.

### *Preparation of tissues*

The animals were killed by a blow on the back of the neck, the liver rapidly removed, weighed and minced in a hand mincer. Routinely 1 g. of this mince was homogenised in 4 ml. of ice cold 0·25 M sucrose using a standard number of strokes of the homogeniser. An aliquot of this 20 per cent w/v homogenate was further diluted with sufficient cold 0·25 M sucrose to give a 7 per cent w/v homogenate. For the experiments in which the tumour was homogenised, it was cut open and, as far as possible, the non-necrotic regions removed with forceps for mincing and homogenising.

Tumours were dissected free of skin and muscle and weighed. The adrenal glands were dissected free from fat and weighed on a 50 mg. torsion balance.

### *Final incubation and extraction conditions*

(a) $\Delta^4$ hydrogenase assay : This was essentially the same as Tomkins (1957). Each incubation tube contained 0·175 $\mu$moles NADP, 2·5 $\mu$moles G–6–P, 20 $\mu$moles Tris-HCl buffer pH 7·4, 0·4 $\mu$mole corticosterone and 0·1 ml. 7 per cent w/v homogenate in 0·25 M sucrose.

In both this and the androstenolone assay, the steroid was added as a solution in 0·02 ml. propylene glycol and the reaction medium and homogenate were preincubated separately for 2 minutes at 37° C. before mixing.

After 10 minutes, incubation at 37° C., the reaction was stopped by the addition of 8 ml. methylene dichloride. The organic and aqueous phases were separated by centrifugation and the protein plus water layer discarded. The methylene dichloride extract was then read at 240 m$\mu$ in a Unicam S.P. 500 spectrophotometer. An extraction control in which the steroid was added just before extraction was carried out with each experiment. The amount of steroid metabolised was obtained by subtracting the amount of steroid remaining after incubation from the extraction control. One unit of $\Delta^4$ hydrogenase activity was defined as a change in optical density of 0·01/minute.

(b) Androstenolone metabolism : Each tube contained 0·3 $\mu$moles NADP, 20 $\mu$moles nicotinamide, 20 $\mu$moles Tris-HCl buffer pH 7·4, 0·5 $\mu$moles androstenolone and 0·2 ml. 20 per cent w/v homogenate in 0·25 M sucrose. After 40

minutes, incubation at 37° C. the reaction was stopped by the addition of 5 ml. methylene dichloride and an aliquot of the methylene dichloride taken to dryness under nitrogen. The androstenolone content was measured by the Pettenkofer reaction. The amount of androstenolone metabolised was calculated by subtracting the amount of steroid remaining after incubation from the extraction control. A calibration graph with androstenolone acetate as standard was prepared for each experiment. One unit of activity was defined as the metabolism of 1 $\mu$g. androstenolone (measured as the acetate)/40 minutes.

All incubations were carried out in duplicate and each result is the mean of 6–8 experiments.

The Pettenkofer reaction was carried out by the method of Munson, Jones, McCall and Gallagher (1948) as modified by Fotherby (1958).

The Blue Tetrazolium reaction was carried out according to Madder and Buck (1952).

Androstenolone was purchased from British Drug Houses Ltd., and crystallised once from ethanol before use. Corticosterone was kindly provided by Professor W. Klyne from the Medical Research Council Steroid Reference Collection. Tris, NAD and dipotassium glucose-6-phosphate were purchased from Sigma Chemical Co. Ltd., St. Louis, Missouri, U.S.A. NADP was purchased from Bochringer and Son Ltd., Mannheim, West Germany. Methylene dichloride was redistilled before use.

### RESULTS

*Rate of tumour growth*

The rate of tumour growth was very variable, depending on whether the tumour adhered to the peritoneal muscle or the skin (Talalay, Takano and Huggins, 1952),

FIG. 1.—Effect of tumour size on liver weight. Mean ± S.E.M.
○ female ● male

FIG. 2.—Effect of tumour size on adrenal weight. Mean ± S.E.M.
○ female ● male but the average time taken to reach a given tumour weight was as follows : 2 g., 7 days ; 5 g., 9 days ; 10 g., 11 days ; 15 g., 13 days ; 20 g., 15 days. Talalay *et al.* (1952) have reported that in rats, the growth rate of Walker 256 implanted intramuscularly is independent of the sex of the host animal.

*Liver size*

Fig. 1 shows the variation of liver weight/100 g. body weight with the weight of the tumour. In the male rats the liver size increase significantly ($p < 0{\cdot}02$)

FIG. 3.—$\Delta^4$ Hydrogenase activity as a function of the weight of homogenate used in the incubations.

in the smallest tumour group but does not increase further once the tumour has reached 10 g. The females appear to be less susceptible to liver enlargement as the weight increase does not become significant ($p < 0{\cdot}05$) until the tumour reaches 10 g. The weight increase is about 10 per cent in both sexes.

Control animals receiving saline injections were killed at varying time intervals up to 20 days after injection but no difference could be found in any of the parameters measured. Accordingly, all of the control results were combined as one group.

*Adrenal size*

Fig. 2 shows the marked sex difference in the response of the adrenal gland to an actively growing tumour. In the female the adrenal size remains constant whereas in the male there is a highly significant ($p < 0{\cdot}01$) 25–40 per cent rise in weight once the tumour reaches 10 g. (5 per cent of the body weight).

$\Delta^4$ *Hydrogenase*

Preliminary experiments with female rat liver were carried out to get the correct conditions for measuring changes of enzyme activity, and Fig. 3 shows that under the conditions used, the enzyme is the rate limiting factor. Although $NADH_2$ can act as hydrogen donor if inorganic phosphate is present (Leybold and Staudinger, 1962 ; McGuire, 1962) this effect is small, so $NADPH_2$, generated from NADP, G–6–P and endogenous G–6–P dehydrogenase was the only pyridine nucleotide used in this assay. NADP concentrations in excess of $\mu$mole/ml. were inhibitory. A concentration of 0·175 $\mu$moles NADP and 0·4 $\mu$moles corticosterone/0·5 ml. final incubation volume was used routinely. Routinely, 7 mg./tissue/incubation tube was chosen for the assay.

It has been reported that the $\Delta^4$ hydrogenase activity of female rat liver is located in the microsome fraction (Forchielli and Dorfman, 1956 ; Tomkins, 1957), but in the experiments reported here there was an appreciable loss of activity if the homogenate was centrifuged at 7000 g for 10 minutes to remove cell debris nuclei and mitochondria. Accordingly whole homogenates were used routinely. This loss of activity is in agreement with the experiments of De Venuto and Westphal (1961) on the metabolism of cortisol by subcellular fractions of rat liver.

It should be emphasized that in using this assay procedure, it is only the " metabolising potential " of the tissue that is being measured as the enzyme has been deliberately saturated with $NADPH_2$ and there is no reason to believe that such a condition prevails *in vivo.*

Fig. 4 and 5 show that $\Delta^4$ hydrogenase activity expressed per g. wet weight of liver and per whole liver/100 g. body weight respectively. Again, a sex difference in the response to tumour growth is evident. In the males the effect is clear cut with a significant rise ($p < 0{\cdot}02$) in enzyme concentration (/g. wet weight) and total hepatic activity when the tumour reaches 5–10 g. In the females the pattern is more complex, but no sustained rise in activity is evident although there is a significant peak of activity ($p < 0{\cdot}02$) expressed on a /g. wet weight basis in the 5–10 g. tumour weight group.

In a few experiments an aliquot of the methylene dichloride extract was used to estimate $\alpha$-ketols by the blue tetrazolium reaction. There was no difference

FIG. 4.—Effect of tumour size on hepatic $\Delta^4$ hydrogenase concentration. Mean ± S.E.M.

○ female ● male

FIG. 5.—Effect of tumour size on total hepatic $\Delta^4$ hydrgenase activity. Mean ± S.E.M.

○ female ● male between the incubated tubes and the extraction controls, so it would appear that under these conditions there is no reduction of the C-20 carbonyl group or removal of the C-17 side chain.

The tumour itself did not show any $\Delta^4$ hydrogenase activity towards corticosterone or androstenedione. This was tested both with 7 mg. and 40 mg. of tumour homogenate/incubation tube.

*Androstenolone metabolism*

Table I shows the pyridine nucleotide requirements. For optimal conditions NADP and nicotinamide are required but not G–6–P. This suggests that the NADP is required in the oxidised form presumably for the oxidation of the $3\beta$ hydroxy group (Kochakian, Carroll and Uhri, 1957) and that there is sufficient $NADPH_2$ available from endogenous substrates for any reductive metabolism such as 7 $\alpha$ (Starka and Kutova, 1962) or 16 $\alpha$ hydroxylation (Colas, 1962).

Fig. 6 shows that under the conditions employed, the enzyme is the rate limiting factor. Routinely, 0·3 $\mu$moles NADP, 0·5 $\mu$moles androstenolone and 40 mg. of tissue were used.

FIG. 6.—Androstenolone metabolism as a function of the weight of homogenate used in the incubations.

TABLE I.—*Pyridine Nucleotide Requirements for the Metabolism of Androstenolone by Rat Liver Homogenates*

All incubation tubes contained 20 $\mu$moles Tris-HCl buffer pH 7·4, 0·5 $\mu$moles androstenolone and 0·2 ml. 20 per cent w/v homogenate in 0·25 M sucrose. Final incubation volume 0·5 ml. The incubations were carried out at 37° for 30 minutes in air.

| Additions | Androstenolone metabolised $\mu$g./g. tissue/hour |
|---|---|
| None . . . . . . . . . | 0 |
| 0·3 $\mu$moles NAD . . . . . . . | 200 |
| 0·3 $\mu$moles NAD + 20 $\mu$moles nicotinamide . | 700 |
| 0·3 $\mu$moles NADP . . . . . . | 600 |
| 0·3 $\mu$moles NADP + 20 $\mu$moles nicotinamide . | 1,200 |
| 0·3 $\mu$moles NADP + 20 $\mu$moles nicotinamide + 2·5 $\mu$moles G–6–P . | 900 |

No significant difference in the disappearance of androstenolone could be detected between the control and tumour-bearing animals (Fig. 7 and 8) other than a slight fall in the livers of female rats bearing very large tumours.

The tumour itself was unable to metabolise androstenolone.

Fig. 7.—Effect of tumour size on hepatic androstenolone metabolising activity. Mean ± S.E.M.
○ female ● male

Fig. 8.—Effect of tumour size on total hepatic androstenolone metabolising activity. Mean ± S.E.M.
○ female ● male

### DISCUSSION

The results presented in this paper do not agree with those published by Goodlad and Clark (1961). Superficially, their results with female rats agree with our results with males in that there was an increase in liver and adrenal size and in total hepatic $\Delta^4$ hydrogenase. However, their results with the $\Delta^4$ hydrogenase could be explained solely by the increase in liver size as the activity/g. wet weight did not change. The experiments reported here indicate an increase in activity/g. wet weight as well as per total liver. They also reported a 30 per cent increase in liver and adrenal weight 30 hours after tumour implantation. This is in marked contrast with the results presented here in which the female adrenal size remained constant throughout the experiment and the male adrenals did not enlarge until the tumour had reached 10 g., about 11 days after implantation.

In reviewing the literature on the Walker 256 tumour there is ample evidence that it does produce adrenal hypertrophy in male rats (Ball and Samuels, 1938 ; Haven, Bloor and Randall, 1949 ; Begg, 1951) but, with the exception of Goodlad and Clark (1961) and a paper by Sure, Theis and Harrelson (1939) in which they do not specify the sex of the animals used to measure adrenal size, no information could be found on the effect of Walker 256 tumour on the adrenals of female rats. It is possible that the difference in results obtained by us and Goodlad and Clark is due to the different experimental conditions used by the two groups, but in view of the dramatic increase in adrenal and liver size obtained by those authors, the increase in liver weight being greater than that obtained after partial hepatectomy, it is felt that the results presented here give a better indication of the events associated with tumour growth than those reported by Goodlad and Clark.

It is interesting that Dalton (1944) found a sex difference in the loss of adrenal lipids in mice with some but not all the tumours he tested. With the tumours that did show a sex difference, the effect on the male adrenals was always larger than on the female. Adams (1959, 1960) has suggested that tumour homogenate injections can counteract the effects of androgens on the intracellular distribution of liver catalase. A similar mechanism may be in operation here since the removal of androgen is known to cause adrenal enlargement in male rats (Yates, Herbst and Urquhart, 1958). It is not clear at what level such antagonism might occur but as the effect of Walker 256 on adrenal size is abolished by hypophysectomy (Ball and Samuels, 1938) it is unlikely to be by direct action on the adrenal gland.

Urquhart, Yates and Herbst (1959) have suggested that the ability of hepatic $\Delta^4$ hydrogenase to inactivate corticosteroids can control the size and output of the adrenal gland via the hypophysis. Thus, any factor influencing the $\Delta^4$ hydrogenase would also influence the adrenal gland. In support of this idea is the present observation that the rise in $\Delta^4$ hydrogenase activity precedes the adrenal weight increase.

With regard to the steroid status of the whole animal, the elevated $\Delta^4$ hydrogenase in males would indicate an elevated secretion and excretion of adrenal corticoids (Urquhart *et al.*, 1959) ; McGuire and Tomkins, 1959 ; Glenister and Yates, 1961), although the question as to whether the enlarged adrenals often seen in tumour-bearing animals are hyper- or hypoactive has not yet been settled (Begg, 1958 ; Claus, Trunnel and Llaurando, 1962 ; Millar, Davis, Toal, Brooks and White, 1962). Nothing is known about the relationship of androstenolone metabolism to adrenal secretion, so no predictions can be made as to the excretion of androstenolone metabolites. Similarly, no predictions can be made about steroid excretion in females so that discussion about a possible relationship of this work to humans with breast cancer is valueless.

SUMMARY

The effect of Walker 256 carcinosarcoma on liver and adrenal weight and hepatic metabolism of corticosterone and androstenolone in male and female rats has been investigated.

The liver weight increased in both sexes whereas adrenal enlargement was only noted in male animals. This enlargement did not become evident until the tumour weight had reached 10 g. (5 per cent of the body weight). In the male rats, the hepatic $\Delta^4$ hydrogenase activity was increased, both on a per g. wet weight and per whole liver basis, before the adrenal enlargement. This would support the idea that the hepatic metabolism of corticosteroids can influence adrenal size and secretion. Because of the high variation in female rats, no clear-cut effect could be detected on $\Delta^4$ hydrogenase activity in these animals.

No significant variation in androstenolone metabolism could be detected in either sex.

The authors are greatly indebted to Dr. G. F. Marrian, F.R.S., Dr. J. Craigie, F.R.S., and Dr. F. C. Chesterman for their help and advice throughout this work.

REFERENCES

Adams, D. H.—(1959) *Brit. J. Cancer*, **13**, 704.—(1960) *Ibid.*, **15**, 386.
Ball, H. A. and Samuels, L. T.—(1938) *Proc. Soc. exp. Biol., N.Y.*, **38**, 441.
Begg, R. W.—(1951) *Cancer Res.*, **11**, 341.—(1958) *Advanc. Cancer Res.*, **5**, 1.
Bulbrook, R. D., Greenwood, F. C. and Hayward, J. L.—(1960) *Lancet*, i, 1154.
Bush, I. E.—(1953) *J. Endocrin.*, **9**, 95.
Claus, G. L., Trunnell, G. B. and Llaurando, G. G.—(1962) *Acta endocr., Copenhagen*. **40**, 584.
Cohen, G. L. and Hume, M.—(1960) *J. clin. Invest.*, **39**, 1584.
Colas, A.—(1962) *Biochem. J.*, **82**, 390.
Craigie, J.—(1949) *Brit. J. Cancer*, **3**, 249.
Dalton, A. J.—(1944) *J. nat. Cancer Inst.*, **5**, 99.
De Venuto, F. and Westphal, V.—(1961) *Biochem. biophys. Acta.*, **54**, 294.
Forchielli, E. and Dorfman, R. I.—(1956) *J. biol. Chem.*, **223**, 443.
Fotherby, K.—(1958) *Biochem. J.*, **69**, 596.
Glenister, D. W. and Yates, F. E.—(1961) *Endocrinology*, **68**, 747.
Goodlad, G. A. D. and Clark, C. M.—(1961) *Brit. J. Cancer*, **15**, 833.
Haven, F. L., Bloor, W. R. and Randall, C.—(1949) *Cancer Res.*, **9**, 511.
Huggins, C. and Dao, T. L. Y.—(1954) *Ann. Surg.*, **140**, 497.
Ketz, H. A., Witt, H. and Mitzner, M.—(1961) *Biochem. Z.*, **334**, 73.
Kochakian, C. D., Carroll, B. R. and Uhri, B.—(1957) *J. biol. Chem.*, **224**, 811.
Kushinsky, D., Kolin, P., Crawley, L. G. and Wu, J.—(1960) *Proc. Amer. Ass. Cancer Res.*, **3**, 127.
Leybold, K. and Staudinger, H.—(1962) *Arch. Biochem.*, **96**, 626.
Loraine, J. A., Strong, J. A. and Douglas, M.—(1957) *Lancet*, ii, 575.
Madder, W. J. and Buck, R. R.—(1952) *Analyt. Chem.*, **24**, 666.
McGuire, J. S.—(1962) *Fed. Proc.*, **21**, 189.
*Idem* and Tomkins, G. M.—(1959) *J. biol. Chem.*, **234**, 791.
Millar, F. K., Davis, J. O., Toal, G. N., Brooks, R. H. and White, G.—(1962) *Proc. Amer. Ass. Cancer Res.*, **3**, 343.
Munson, P. L., Jones, M. E., McCall, P. J. and Gallagher, T. F.—(1948) *J. biol. Chem.*, **176**, 73.

STARKA, L. AND KUTOVA, J.—(1962) *Biochim. biophys. Acta.*, **56**, 76.
SURE, B., THEIS, R. M. AND HARRELSON, R. T.—(1939) *Amer. J. Cancer*, **36**, 252.
SWEAT, M. L. AND BRYSON, M. J.—(1960) *Biochim. biophys. Acta*, **44**, 217.
TALALAY, P., TAKANO, G. M. V. AND HUGGINS, C.—(1952) *Cancer Res.*, **12**, 834.
TOMKINS, G. M.—(1957) *J. biol. Chem.*, **225**, 13.
URQUHART, J., YATES, F. E. AND HERBST, A. L.—(1959) *Endocrinology*, **64**, 816.
VAN DE WIELE, R. AND LIEBERMAN, S.—(1960) In 'Biological Activities of Steroids in Relation to Cancer'. Edited by Pincus, G. and Vollmer, E. P. New York (Academic Press Inc.), p. 93.
YATES, F. E., HERBST, A. L. AND URQUHART, J.—(1958) *Endocrinology*, **63**, 887.